# Extending the evidence base for eating disorders prevention: the impact of a dissonance-based intervention on positive body image, intuitive eating and self-objectification

**DOI:** 10.1186/2050-2974-2-S1-O38

**Published:** 2014-11-24

**Authors:** Phillippa C Diedrichs, Emma Halliwell, Nicole Paraskeva

**Affiliations:** 1 Centre for Appearance Research, University of the West of England, Bristol, United Kingdom

## 

Recently, there have been significant advances in eating disorders (ED) prevention. Cognitive dissonance programs have demonstrated reductions in ED risk factors outlined in the dual pathway model (Stice, 1994) among university-age women in the USA and the UK. However, self-objectification theory proposes additional empirically supported risk-factors for EDs. In addition, body image research has recently expanded its focus to consider the promotion of positive body image. The present study examines the impact of a dissonance-based intervention on self-objectification, positive body image, and intuitive eating. Sixty-nine British women (M=19.06 years) took part in the intervention as part of their undergraduate psychology coursework. A separate sample of 47 undergraduate women formed a non-randomized control group. Baseline measures typically used to evaluate the intervention were administered alongside measures of self-objectification, positive body image, intuitive eating and life satisfaction pre- and immediate post- intervention, and at 8-week follow-up. As hypothesised, the intervention led to significant improvements relative to the control group in body dissatisfaction, dietary restraint, surveillance, body shame, body appreciation and intuitive eating at post-intervention and 8 week follow-up. It also improved life satisfaction at post-intervention only. The results suggest that dissonance-based interventions have benefits beyond risk factors identified in the dual-pathway model.

This abstract was presented in the **Prevention & Public Health** stream of the 2014 ANZAED Conference.

